# Peptide-Mucin Binding and Biosimilar Mucus-Permeating Properties

**DOI:** 10.3390/gels8010001

**Published:** 2021-12-21

**Authors:** Xiaohong Sun, Raliat O. Abioye, Ogadimma D. Okagu, Chibuike C. Udenigwe

**Affiliations:** 1 School of Nutrition Sciences, Faculty of Health Sciences, University of Ottawa, Ottawa, ON K1H 8M5, Canada; 2 College of Food and Biological Engineering, Qiqihar University, Qiqihar 161006, China; 3 Department of Chemistry and Biomolecular Sciences, University of Ottawa, Ottawa, ON K1N 6N5, Canada; rabio069@uottawa.ca (R.O.A.); ookag095@uottawa.ca (O.D.O.)

**Keywords:** mucin-binding activity, biosimilar mucus-permeating property, in vitro model, mucin binding-mucus permeability relation, hydrophobicity index, bioavailability

## Abstract

This study aimed to understand the role of the mucus layer (a biological hydrogel) in the transport mechanisms of peptides. Using established in vitro models, the mucin-binding activity and mucus-permeating property of peptides were determined. Uncharged peptides with relatively high hydrophilicity, including MANT, TNGQ, and PASL, as well as cationic peptides, including KIPAVF and KMPV, possessed strong mucin-binding activity. Contrarily, uncharged peptides with high hydrophobicity index, including YMSV and QIGLF, exhibited weak mucin-binding activity. Only TNGQ, which has high Boman index and hydrophilicity, showed a high biosimilar mucus-permeating property with a permeability of 96 ± 30% after 60 min. TNGQ showed the potential for high bioavailability due to the high mucin-binding and biosimilar mucus-permeating activities.

## 1. Introduction

Bioactive peptides, usually consisting of 2–20 amino acid residues, are commonly released from the parent proteins by enzymatic hydrolysis or microbial fermentation [1]. In vitro and in vivo studies have demonstrated that bioactive peptides exert health-promoting properties, including antihypertensive, antioxidant, anti-inflammatory, immunomodulatory, antidiabetic, and anticancer activities [1,2]. However, there are some contradictions in the reported in vitro and in vivo efficacies of bioactive peptides, possibly due to the low oral bioavailability or over-simplicity of the experimental procedures [3].

**Figure 1:**
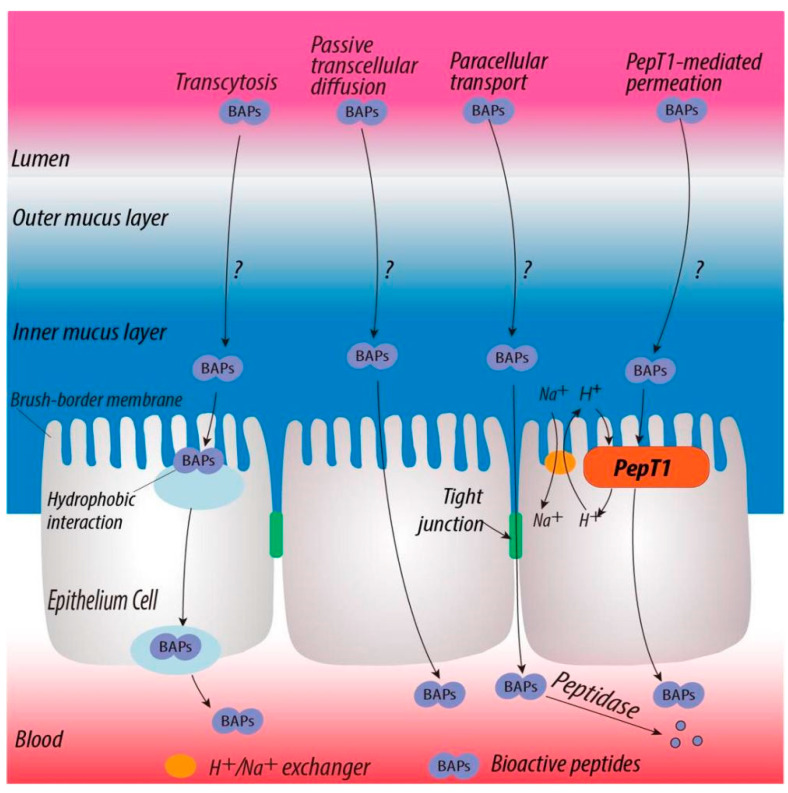
Plausible pathways for the transport of bioactive peptides across the gastrointestinal epithelium into the blood circulation, including PepT1-mediated permeation, paracellular transport via tight junctions, transcytosis, and passive transcellular diffusion. It is still not known how peptides interact with mucin and penetrate the mucus layers before transporting across the underlying epithelium. This figure was reprinted from [5] with permission from Elsevier.

To enhance the bioavailability of bioactive peptides, their transport mechanisms have been investigated using the Caco-2 cell line as a model. It has been reported that peptides are transported across the intestinal epithelium into blood circulation through four distinct pathways, including PepT1-mediated permeation, paracellular transport via tight junctions, transcytosis, and passive transcellular diffusion, as shown in Figure 1 [4,5]. However, the Caco-2 cell line lacks the mucus layer that is located on the surface of the epithelial membrane and functions as a protective barrier [6]. Furthermore, mucus acts as a selectively permeable layer for the diffusion and absorption of biomolecules or nutraceuticals in the gastrointestinal tract, which would impact their bioavailability [7].

Mucus is a biological hydrogel that constitutes of approximatively 95% water, 1~2% lipids, electrolytes, proteins, and 1~5% mucins [7]. Mucins are the major functional components of mucus that are responsible for the viscoelastic properties of the mucus layer [7]. Mucins are glycoproteins consisting of 50~80% (*w*/*w*) carbohydrates, which are primarily composed of N-acetylgalactosamine (GalNAc), N-acetylglucosamine (GlcNAc), fucose, galactose, mannose, and sialic acid [8]. Mucins carry a net negative charge at neutral pH due to the presence of terminal sialic acid and sulfate groups [9]. The oligosaccharide chains commonly attached to the linear mucin protein backbone, through α-1-glycosidic bonds from GalNAc to the hydroxyl side chains of Ser and Thr, results in a “bottle brush” configuration [7,10]. Mucin monomers can further dimerize and polymerize through the formation of C-terminal disulfide bonds [7].

Co-culture of Caco-2 cells with mucus-secreting goblet cells (e.g., Caco-2 and HT29 co-culture monolayers) has been used as a more physiologically relevant model to evaluate the transport of bioactive peptides [11]. To date, it is still not well understood how peptides interact with mucin and penetrate the mucus layers before transporting across the underlying epithelium, as illustrated in Figure 1 [5]. Thus, the objectives of this study were to (i) understand the peptide structure-mucin binding relationship, (ii) evaluate biosimilar mucus-permeating property of peptides, and (iii) correlate mucin-binding property with mucus permeability of peptides using in vitro models.

## 2. Materials and Methods

### 2.1. Materials and Chemicals

Seven unmodified peptides with different physicochemical properties (i.e., net charge, hydrophobicity index, and Boman index) were selected as model peptides and synthesized by GenScript (Piscataway, NJ, USA). In this study, the hydrophobicity index estimated the relative hydrophobicity of a peptide and the Boman index indicated the binding potential of a peptide to a protein receptor; peptide information is presented in Table 1. Lectin from Triticum vulgaris (wheat germ agglutinin, WGA, FITC conjugate), mucin from porcine stomach (type III, bound sialic acid 0.5~1.5%), L-α-phosphatidylcholine, poly(acrylic acid) (PAA), linoleic acid, Tween^®^ 80, o-phthaldialdehyde (OPA), Tween^®^ 20, bovine serum albumin (BSA, 96% purity), cholesterol, sodium phosphate monobasic, sodium phosphate dibasic, sodium chloride, sodium dodecyl sulfate (SDS), and β-mercaptoethanol were purchased from Millipore Sigma (Burlington, MA, USA). Milli-Q water was obtained using the Milli-Q water supply system (Millipore Corporation, Billerica, MA, USA) with a total organic carbon level ≤5 ppb and resistivity of 18.2 MΩ·cm at 25 °C.

### 2.2. Physicochemical Properties of Peptides

Physicochemical properties of the peptides, including isoelectric point (pI), net charge, hydrophobicity index, and Boman index, were calculated using the Peptides package in R. pI was calculated using the EMBOSS scale; net charge at pH 7.0 was calculated using the Bjellqvist scale; and hydrophobicity index was calculated using the KyteDoolittle scale.

### 2.3. Determination of Mucin-Binding Activity of Peptides

**Figure 2:**
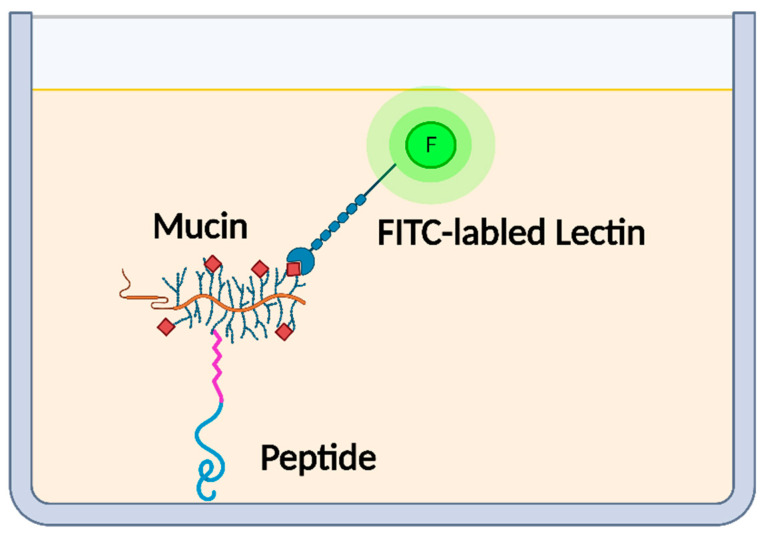
Schematic diagram of peptide-mucin binding assay (Image created with BioRender).

Peptide-mucin binding assay was established according to the specific interaction between porcine gastric mucin and wheat germ agglutinin, as illustrated in Figure 2. Briefly, the peptide solution (1 mM) in phosphate buffered saline (5 mM) containing NaCl (150 mM) at pH 7.2 was coated on a Nunc MaxiSorp™ flat-bottom 96-well microplate overnight at room temperature. After washing with PBS containing 0.05% Tween 20 (PBST) to remove free peptides, the wells were blocked with 2% (*w*/*v*) BSA solution in PBS buffer for 1 h at 37 °C. Mucin sample, including whole mucin at the concentration of 10 mg/mL in PBS or its supernatant (soluble mucin), was applied to each well after washing out the free BSA, which was previously incubated for 24 h at 37 °C. Mucin supernatant was collected by centrifugation at 2935× *g* for 30 min (Eppendorf 5430R, Hamburg, Germany) and subsequently subjected to a sterile polyethersulfone membrane syringe filter (VWR International, West Chester, PA, USA) with a pore size of 0.22 µm. Next, the unbounded mucin was washed out using PBST, and FITC-labeled wheat germ agglutinin (FITC-WGA, 25 μg/mL) in PBS was added and incubated for 30 min at room temperature. Lastly, the amount of FITC-WGA binding to mucin was measured using the Spark multimode microplate reader (Tecan, Stockholm, Sweden) at λ_ex_ and λ_em_ of 494 and 518 nm, respectively. The mucin-binding activity of peptides was estimated using Equation (1).

(1) $$Mucin\;binding\;activity=\frac{F}{F_{0}}$$

*F* is the fluorescence intensity of the sample assay (with peptides) or positive control (FITC-WGA at 25 μg/mL) and *F*_0_ is the fluorescence intensity of the negative control (without peptides).

### 2.4. Quantification of Biosimilar Mucus-Permeating Property of Peptides

An in vitro cell-free model was established in this study for the quantification of mucus-permeating property of peptides. Biosimilar mucus used in the model consisted of 0.3% (*w*/*v*) PAA, 5% (*w*/*v*) mucin, 3.1% (*w*/*v*) BSA, 0.6% (*w*/*v*) lipid mixture including 0.06% (*w*/*v*) linoleic acid, 0.36% (*w*/*v*) cholesterol, 0.18% (*w*/*v*) phosphatidylcholine, and Tween 80 (25% of lipid mixture, *w*/*w*) [12]. First, the lipid was mixed with Tween 80, whereafter PBS buffer with 150 mM NaCl (5 mM, pH 7.2) was slowly added under magnetic stirring (Cimarec, ThermoFisher Scientific, Waltham, MA USA). On the other hand, PAA was dissolved in PBS buffer prior to the addition of mucin under stirring, and pH adjusted to 7.0 using NaOH (1 M). After mixing the lipids and mucin suspension, BSA was added to finalize the biosimilar mucus and the pH was adjusted to 7.4. The mucus mixture was stored overnight at 4 ℃ before dialyzing (Fisherbrand Regenerated Cellulose Dialysis Tubing, MWCOs from 3500 to 14,000 Daltons) for 72 h against PBS buffer (5 mM, pH 7.2) containing 150 mM NaCl to remove unbound lipids and contaminating proteins from BSA. Mucus-permeating assay was conducted by firstly adding 3.8 mL of dialyzed mucus suspensions to the filter of the centrifugal unit (4 mL, 10 KDa cutoff, Amicon Ultra, Millipore Sigma) followed by centrifugation at 2935× *g* for 15 min to make the mucus settle down and completely cover the entire membrane before adding peptide solutions (1 mM, 1 mL) or 1 mL of PBS buffer as blank. Afterwards, the permeate during centrifugation at 25 ℃ and 470× *g* was collected every 15 min for 4 h. The peptide content in the permeate was measured using the OPA method [13]. The mucus permeability of the peptides was quantified using Equation (2).

(2) $$P=\frac{n_{p}-n_{b}}{n_{T}}\times 100$$

*PP* is the percentage permeability, *n_p_* represents the permeated peptides (µmol), *n_b_* is the blank (µmol), and *n_T_* is the total loaded peptide amount (µmol).

### 2.5. Statistical Analysis

All mucin-binding and biosimilar mucus-permeating activities were evaluated at least three times, while the assays were further completed in triplicate. The results were expressed as mean ± standard error of the mean (SEM). Statistical analysis was performed by one-way analysis of variance using IBM SPSS statistics 19 (SPSS Inc., Chicago, IL, USA). Significant differences were defined at a 5% level (*p* < 0.05).

## 3. Results and Discussion

### 3.1. Mucin-Binding Activity of Peptides

**Figure 3:**
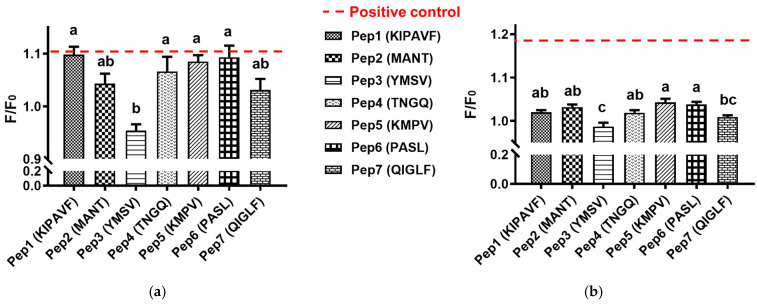
(**a**) Soluble mucin-binding activity of peptides; positive control value was 1.100 ± 0.019. Different letters indicate significantly different mean values (*p* < 0.05); (**b**) Whole mucin-binding activity of peptides; positive control value was 1.175 ± 0.028. Different letters indicate significantly different mean values (*p* < 0.05).

Mucin being a component of mucus in the gastrointestinal tract helps in regulating the penetration of biomolecules into the epithelial membrane. The nature and strength of its interaction with nutraceutical agents affect the permeability and hence bioavailability of nutrients. For this reason, the mucin binding activity of peptides was investigated using a soluble fraction of porcine gastric mucin (less viscous) and whole mucin (more viscous) to simulate the outer and inner mucus layer, respectively. As shown in Figure 3a, the F/F_0_ values differed for most of the peptides, signifying different extents of binding to mucin. YMSV showed the lowest binding to soluble mucin compared to KIPAVF, TNGQ, KMPV, and PASL (*p* < 0.05), while YMSV had similar mucin-binding property to that of MANT and QIGLF (*p* > 0.05). Likewise, YMSV showed the lowest binding activity to whole mucin (*F*/*F*_0_ 0.987 ± 0.009), which was not significantly different from that of QIGLF (*F*/*F*_0_ 1.009 ± 0.004, *p* > 0.05) in Figure 3b. Therefore, five peptides (KIPAVF, MANT, TNGQ, KMPV, and PASL) demonstrated a relatively higher mucin-binding activity to both soluble and whole mucin when compared to YMSV and QIGLF.

As shown in Figure 3 and Table 1, uncharged peptides with relatively high hydrophilicity, including MANT, TNGQ, and PASL, showed strong binding activity to both soluble and whole mucin. The Boman index showed that only TNGQ had a high protein-binding potential, as predicted based on the solubility properties of amino acid side-chains. Collectively, the results were in line with the mucoadhesive property of hydrophilic polymers, which were usually involved in hydrogen bonding and electrostatic interactions [14,15]. On the contrary, uncharged peptides with high hydrophobicity index, including YMSV and QIGLF, showed the lowest binding to soluble and whole mucins. QIGLF had the lowest Boman index, which supported the in vitro mucin-binding result. Moreover, cationic KIPAVF and KMPV exhibited strong binding activity to the mucins. This binding was possibly driven by the electrostatic interaction between the N-terminal lysine residue of the peptides and highly anionic surface of mucins (e.g., the anionic sialic acid and sulphonated residues), as reported for cationic antimicrobial peptides [16,17]. Therefore, peptide-mucin binding was likely governed by electrostatic interactions.

### 3.2. Mucus-Permeating Property of Peptides

**Figure 4:**
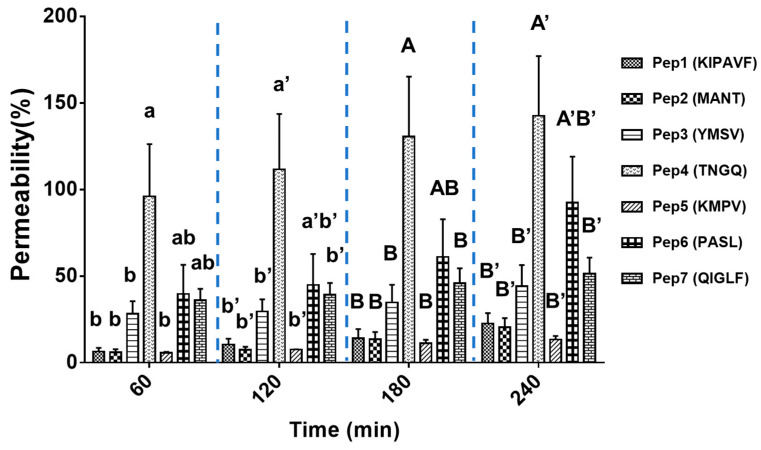
Biosimilar mucus-permeating property of peptides at 60, 120, 180, and 240 min that was quantified by a simple and efficient in vitro cell-free model established in this study. One-way analysis of variance was performed within each time point, and different letters indicate significantly dif-ferent mean values (*p* < 0.05).

The in vitro cumulative mucus permeability of the peptides at 60, 120, 180, and 240 min is shown in Figure 4. At 60 min, TNGQ had the highest mucus-permeating property, which reached 96 ± 30%. This was followed by PASL with a mucus permeability of 40 ± 17%. The trend of mucus permeability did not change throughout the 240-min period. The final mucus permeability for the peptides (1–7) at 240 min were 23 ± 6%, 21 ± 5%, 44 ± 12%, 142 ± 35%, 13 ± 2%, 92 ± 27%, and 51 ± 9%, respectively. The mucus permeability of TNGQ was more than 100%, possibly due to the impurity of BSA in the biosimilar mucus that was not completely removed during dialysis, as well as the detection of total peptides rather than the targeted peptides by the OPA method. The highest mucus-permeating property of TNGQ may have been attributed to its net zero charge and highly hydrophilic property (Table 1). This property was comparable to that of polyethylene glycol (PEG), which was commonly used to promote mucus permeation due to its hydrophilic and electroneutral nature [18].

Furthermore, mucin-binding activity of the peptides did not correlate with their mucus-permeating properties. For instance, YMSV and QIGLF, with uncharged and highly hydrophobic features, showed weak mucin-binding activity and low mucus permeability. In this case, the peptides would be transported along the surface of the mucus layer without interaction or permeation. TNGQ and PASL showed strong mucin-binding activity and a high mucus permeability. This indicated a temporary binding and release mechanism that would favor peptide uptake. Lastly, KIPAVF, MANT, and KMPV had strong mucin-binding activity and a low mucus permeability. This was due to the attraction forces that held the peptides and mucin together preventing peptide dissociation from the complex for the entire assay duration. The mucin-binding activity of peptides could increase their residence time at the intestinal absorption sites. However, extremely high mucin-binding activity would cause peptides to be trapped in the mucus layer, thus reducing subsequent penetration and transit to the apical domain of enterocytes [19]. Therefore, peptides with strong mucin-binding activity combined with high mucus permeability, such as TNGQ and PASL, presented a strong potential for high bioavailability towards intestinal uptake.

Mucus-permeating property of the peptides was quantified using an in vitro cell-free model established in this study and OPA assay, which were simple, efficient, and cost-effective, but presented some limitations. For example, the commercially available porcine gastric mucin could not form gel layers, making the model less physiologically relevant in simulating mucus thickness. Possible contamination from BSA in the biosimilar mucus could have influenced the quantification of mucus-permeating property. The results of mucus-permeating property showed wide variations; therefore, high-performance liquid chromatography could be considered in the future for quantifying the targeted peptide. Moreover, a larger peptide library with diverse structural and physicochemical properties needs to be investigated. For example, the effects of the cationic lysine residue position within the peptide sequence on the mucin-binding and mucus-permeating properties will be of particular interest.

## 4. Conclusions

In this study, an in vitro model was established and used to determine the mucin-binding and mucus-permeating activities of peptides. Results demonstrated that uncharged peptides with relatively high hydrophilicity and cationic peptides showed strong mucin-binding activities, whereas uncharged peptides with high hydrophobicity index exhibited weak mucin-binding activity. The uncharged and highly hydrophilic peptide, TNGQ, possessed the highest mucus-permeating property. However, the mucin-binding activities of the peptides did not correlate with their mucus-permeating properties. Overall, strong mucin-binding activity and high mucus permeability should increase the bioavailability of peptides. This study provides important insight on the role of peptide structure on their transport mechanisms from the mucus perspective.

## Figures and Tables

**Table 1:** Peptide information and calculated physicochemical properties.

No.	Sequence	Purity (%)	MW	pI	Net Charge at pH 7.0	Hydrophobicity Index	Boman Index
Pep1	KIPAVF	98.7	673.8	9.70	+0.9977	1.300	−1.36
Pep2	MANT	98.7	435.5	6.10	−0.0020	−0.125	1.26
Pep3	YMSV	95.5	498.6	6.10	−0.0029	1.000	−0.71
Pep4	TNGQ	95.7	418.4	6.10	−0.0020	−2.025	3.45
Pep5	KMPV	99.2	473.6	9.70	+0.9977	0.150	−0.21
Pep6	PASL	99.2	386.4	6.10	−0.0020	0.800	−0.83
Pep7	QIGLF	98.1	576.7	6.10	−0.0020	1.440	−1.64

## Data Availability

Data supporting the findings are available within the article.
